# A Comparative Study of Melittins from *Apis florea* and *Apis mellifera* as Cytotoxic Agents Against Non-Small Cell Lung Cancer (NSCLC) Cells and Their Combination with Gefitinib

**DOI:** 10.3390/ijms26062498

**Published:** 2025-03-11

**Authors:** Pachara Sattayawat, Thida Kaewkod, Saruda Thongyim, Chutipa Chiawpanit, Yupanun Wutti-in, Chutamas Thepmalee, Yingmanee Tragoolpua, Terd Disayathanoowat, Aussara Panya

**Affiliations:** 1 Department of Biology, Faculty of Science, Chiang Mai University, Chiang Mai 50200, Thailand; pachara.sattayawat@cmu.ac.th (P.S.); thida.kaewkod@cmu.ac.th (T.K.); saruda.th@cmu.ac.th (S.T.); chutipa.c@cmu.ac.th (C.C.); yingmanee.t@cmu.ac.th (Y.T.); terd.dis@cmu.ac.th (T.D.); 2 Cell Engineering for Cancer Therapy Research Group, Faculty of Science, Chiang Mai University, Chiang Mai 50200, Thailand; yupanun.wuttiin@cmu.ac.th; 3 Research Center of Deep Technology in Beekeeping and Bee Products for Sustainable Development Goals (SMART BEE SDGs), Faculty of Science, Chiang Mai University, Chiang Mai 50200, Thailand; 4 Office of Research Administration, Chiang Mai University, Chiang Mai 50200, Thailand; 5 Department of Medical Technology, Faculty of Associated Medical Sciences, Chiang Mai University, Chiang Mai 50200, Thailand; 6 Division of Biochemistry, School of Medical Sciences, University of Phayao, Phayao 56000, Thailand; chutamas.th@up.ac.th

**Keywords:** anti-cancer peptides, melittin, NSCLC, bee venom, EGFR

## Abstract

Lung cancer remains one of the most lethal malignancies, often diagnosed at advanced stages, leading to limited treatment options. Thus, identifying natural products with potent anti-cancer activity is crucial for improving treatment outcomes. While the melittin from *Apis mellifera* (Mel-AM) has been extensively studied, the melittin from *Apis florea* (Mel-AF), a bee species native to Southeast Asia, remains relatively underexplored. These peptides were comparatively investigated against non-small cell lung cancer (NSCLC) cell lines, A549, NCI-H460, and NCI-H1975. Mel-AF demonstrated a superior cytotoxicity (cytotoxic concentration 50% (CC50) = 2.55–5.06 µg/mL) compared to Mel-AM (CC50 = 4.32–8.48 µg/mL). While both peptides induced apoptosis via the intrinsic mitochondrial pathway, Mel-AF exhibited a more pronounced effect, significantly enhancing apoptosis induction compared to Mel-AM. Both peptides inhibited cell migration and invasion; however, when combined with gefitinib, Mel-AF more effectively enhanced the drug’s inhibitory effects on the A549 and NCI-H460 cell lines compared to Mel-AM, underscoring its superior potential as a therapeutic agent. Altogether, we demonstrated that these peptides induced apoptosis in NSCLC cell lines, with Mel-AF having more pronounced effects, and the combination use of peptides with a chemotherapeutic drug showed synergistic effects against lung cancer cells, enhancing their practical use in lung cancer treatments.

## 1. Introduction

While smoking behavior remains the primary cause of lung cancer, concerns regarding air pollution, particularly the role of particulate matter (PM) in lung cancer, have been raised. Recently, with lung cancer being one of the leading causes of death in Thailand [1], PM2.5 has become a critical issue, particularly in the northern region [2,3]. Generally, lung cancers are classified into the following two types: small-cell lung cancer (SCLC), with an incidence of about 15%, and non-small cell lung cancer (NSCLC), with an incidence of about 85% [4]. Depending on the stage of the diagnosed cancer, treatments for lung cancer may involve surgery, radiation therapy, chemotherapy, or targeted therapy. About 70% of NSCLC patients are diagnosed when the cancer has advanced to stage III, which may result in an inability to undergo surgery [5]. In Thailand, the overall 5-year survival rate for NSCLC is around 24%, and if the survival rates based on treatment means and stages are considered, the 5-year survival rate for patients who undergo surgery as a primary treatment for stage I NSCLC is around 63%, whereas, for stage II and III, the 5-year survival rate with combined treatments (chemotherapy, radiation therapy, and sometimes surgery) is about 35%. For advanced or metastatic NSCLC (stage IV), the 5-year survival rate with systemic treatments like chemotherapy, targeted therapy, and immunotherapy is lower, typically around 7% [1]. This urges the development of other means for lung cancer treatments.

Exploring natural products with cytotoxic properties against cancer cells presents a promising avenue for the discovery of cancer therapeutics. Bee venom is naturally synthesized by bees. It is odorless and transparent, containing several bioactive compounds, with melittin constituting a main component. Regarding its medicinal uses, melittin has been demonstrated to have anti-viral [6], anti-microbial [7], and particularly anti-inflammatory [8,9] effects, where chronic inflammation may lead to cancer cell proliferation [10]. While Mel-AM, the melittin peptide from *Apis mellifera* (the European honeybee), has been extensively studied and shown to induce apoptosis and inhibit migration and invasion in non-small cell lung cancer (NSCLC) cells [11,12,13], research on Mel-AF, the melittin peptide from *Apis florea* (the dwarf honeybee), remains limited. *A. mellifera* is a domesticated species commonly found across Europe and can be kept in managed environments worldwide. In contrast, *A. florea* is native to Southeast Asia, including Thailand, and typically lives in natural habitats such as forests, making it less accessible and less studied. Due to the smaller colony size of *A. florea* compared to *A. mellifera*, it is feasible that its melittin has evolved to compensate with a greater potency, enhancing its defensive efficacy and cytotoxicity. This gap highlights the need for more comparative studies to explore the potential advantages of Mel-AF in comparison with Mel-AM. Moreover, the use of natural products in combination with chemotherapeutic drugs has always been of interest to subside the side effects associated with the latter [14]. In the case of melittin, one study demonstrated synergistic effects by combining melittin with sorafenib, a chemotherapeutic drug, in HepG2 cells [15].

In this present work, melittin peptides from two bee species, *A. florea* (Mel-AF) and *A. mellifera* (Mel-AM), were evaluated for their ability to inhibit the viability of A549, NCI-H460, and NCI-H1975 lung cancer cells. To our knowledge, this is the first study investigating a melittin peptide from *A. florea* in NSCLC cells. Comparative effects between melittin peptides from *A. florea* and *A. mellifera* were, therefore, evaluated. Cellular apoptosis and the expression of proteins associated with apoptosis resulting from melittin treatment were investigated along with the inhibition of cell migration and invasion abilities. Moreover, the combination use of melittin peptides with gefitinib, a chemotherapeutic drug, was further investigated to explore the most suitable use of these peptides. The overall findings of this work demonstrated the potential of both melittin peptides as cytotoxic agents, with variations observed between the two bee species. Synergistic effects were observed with both melittin peptides and gefitinib, as their combination enhanced cytotoxic activity, suggesting potential applications in cancer treatments.

## 2. Results

### 2.1. Effect of Melittin Peptides on Lung Cancer Cell Viability

**Figure 1:**
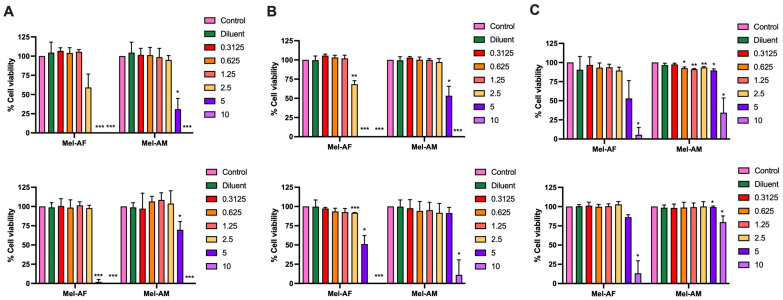
Cytotoxicity of melittin peptides on lung cancer cells. Three lung cancer cell lines: (**A**) A549, (**B**) NCI-H460, and (**C**) NCI-H1975 were treated with the melittins from *A. florea* (Mel-AF) and *A. mellifera* (Mel-AM) at 0, 0.3125, 0.625, 1.25, 2.5, 5, and 10 µg/mL. All treated cells were incubated for 24 h (top) and 48 h (bottom). Data are presented as mean ± SD values of at least three independent replicates. Asterisk (*) indicates significance at *p* ≤ 0.05 (*), *p* ≤ 0.01 (**), and *p* ≤ 0.001 (***).

To investigate the cytotoxic effects of melittin peptides from two different bee species, Mel-AF from *A. florea* and Mel-AM from *A. mellifera*, on the viability of lung cancer cells, different concentrations of both peptides were used to treat the following three lung cancer cell lines: A549, NCI-H460, and NCI-H1975 compared to Vero cells as a non-cancerous cell line. It was shown that Mel-AF was more toxic to all cell lines compared to Mel-AM. The cytotoxic concentration 50% (CC50) values after 24 h of treatment of Mel-AF in A549, NCI-H460, and NCI-H1975 cells were 2.55, 2.61, and 5.06 µg/mL, respectively, while they were 4.32, 5.10, and 8.48 µg/mL in the case of Mel-AM (Supplementary Table S1). At high concentrations (5 and 10 µg/mL), Mel-AF and Mel-AM exhibited significant or complete inhibitory effects on all lung cancer cell lines (Figure 1). Interestingly, A549 was shown to be most sensitive to both peptides at 24 h, followed by NCI-H460 and NCI-H1975, respectively (Figure 1 (top)). A similar trend was also observed at 48 h after treatment (Figure 1 (bottom)). Moreover, in our study, Vero cells as a non-cancerous cell control were shown to be less impacted by Mel-AF (10 µg/mL at 48 h), and Mel-AM did not show inhibition at the same concentrations and times of treatment (Supplementary Figure S1). This potentiates their use, as they are relatively selective toward cancer cells.

### 2.2. Apoptosis Induction by Melittin Peptides

**Figure 2:**
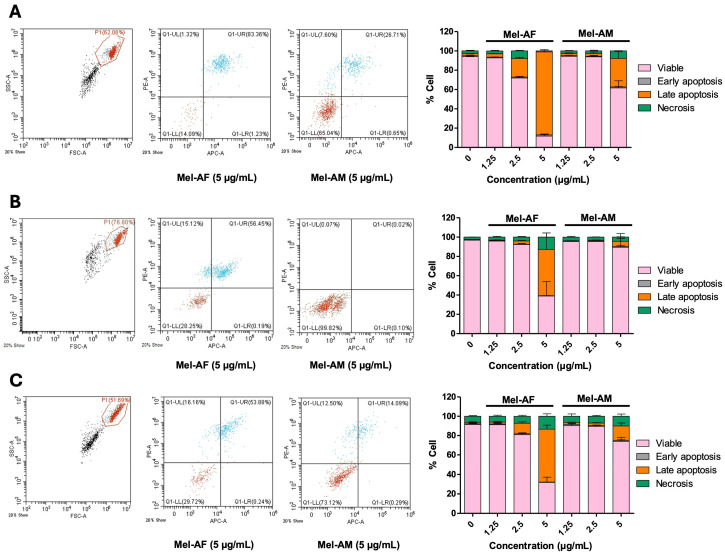
Effect of melittin peptides on cell apoptosis. (**A**) A549, (**B**) NCI-H460, and (**C**) NCI-H1975 cells were treated with 1.25, 2.5, and 5 µg/mL of melittin peptides: Mel-AF and Mel-AM, for 4 h and were subjected to flow cytometric analysis. The bar graphs represent percentages of viable, early apoptotic, late apoptotic, and necrotic cells in each treatment. The intact cancer cell population was gated (left) as designated P1 and discriminated for living cell population (light and dark brown dots) and dead cell population (blue dots). Data are presented as mean ± SD values of at least three independent replicates.

A melittin peptide was previously reported to induce cell death in A549 and NCI-H460 cells via apoptosis [13]. In this study, flow cytometry was used to assess apoptosis in all three cell lines after 4 h of treatment with varying concentrations (1.25, 2.5, and 5 µg/mL) of Mel-AF and Mel-AM. The results suggested that, at equivalent concentrations, Mel-AF treatment induced higher levels of apoptotic cell death compared to Mel-AM, as shown in Figure 2A–C. The trend was similar in all cell lines, but it was noticeable that the apoptosis of A549 was more pronounced compared to the other cell lines, which is in agreement with the cytotoxicity results. The percentages of apoptotic cells at 5 µg/mL of Mel-AF were 87.14 ± 2.32%, 48.35 ± 16.96%, and 54.81 ± 4.26% in A549, NCI-H460, and NCI-H1975, respectively, whereas the percentages of apoptotic cells at 5 µg/mL of Mel-AM were 30.58 ± 7.24%, 6.23 ± 5.33%, and 15.97 ± 3.29% in A549, NCI-H460, and NCI-H1975, respectively. To further explore the apoptosis pathways, Western blot analysis of procaspase 9, procaspase 8, procaspase 3, and Bcl-2 was performed after 2 h of treatment. The results revealed lower expression levels of procaspase 9 and procaspase 3. However, no significant differences were observed in the expression levels of the other proteins (Supplementary Figure S2). This suggests that melittin peptides may induce apoptosis through the intrinsic mitochondrial pathway.

### 2.3. Effect of Melittin Peptides on Cell Migration and Invasion Abilities

**Figure 3:**
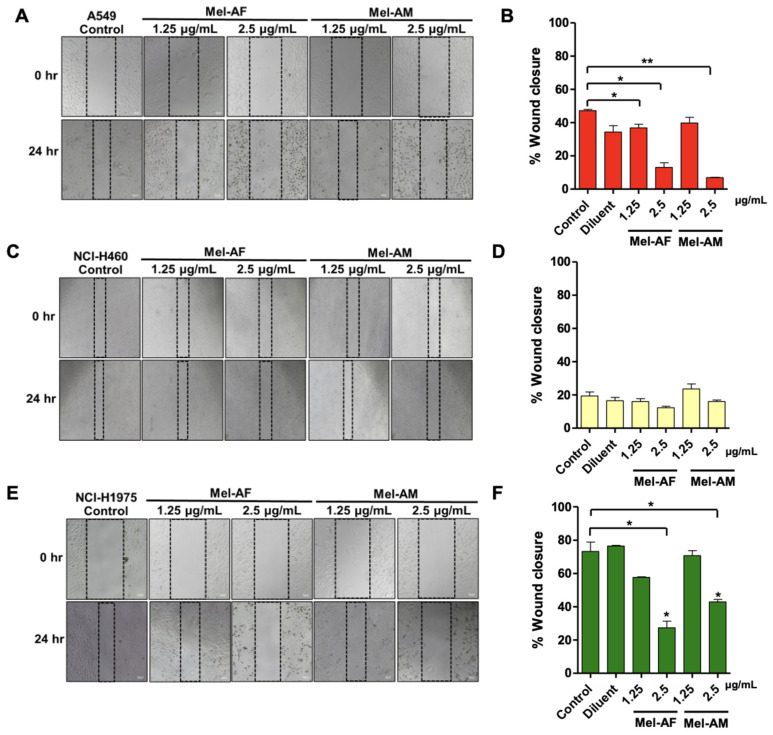
Effect of melittin peptides on cell migration ability. Suppression of cell migration ability by Mel-AF and Mel-AM on different lung cancer cell lines: (**A**,**B**) A549, (**C**,**D**) NCI-H460, and (**E**,**F**) NCI-H1975 was demonstrated using scratch wound assay when compared with untreated cells (Control). (**A**,**C**,**E**) Images depicting wounds at 0 and 24 h after treatment with Mel-AF and Mel-AM are shown. Data are presented as mean ± SD values of at least three independent replicates. Asterisk (*) indicates significance at *p* ≤ 0.05 (*) and *p* ≤ 0.01 (**).

The migration and invasion abilities of lung cancer cells play crucial roles in the formation of metastasis, making them significant targets for potential therapeutic interventions [16]. The effects of Mel-AF and Mel-AM on these cell migration and invasion abilities were investigated using the scratch wound assay and the transwell invasion assay, respectively. The scratch wound assay suggested several points. Firstly, both peptides demonstrated a greater suppression of A549’s migration ability compared to NCI-H460 and NCI-H1975 (Figure 3), as A549 was the most sensitive cell line. Mel-AF at concentrations of 1.25 and 2.5 µg/mL was shown to significantly suppress A549 cell migration at the % wound closure of 36.91 ± 3.64% and 13.03 ± 4.87%, respectively, compared to the control at 47.24 ± 1.17%. Mel-AM was also shown to inhibit A549 cell migration at 2.5 µg/mL with 6.93 ± 0.31% wound closure. Similarly, in the case of NCI-H1975, a concentration of 2.5 µg/mL of both Mel-AF and Mel-AM could also suppress cell migration with 27.33 ± 6.95% and 42.96 ± 2.56% wound closure, respectively, compared to 73.27 ± 9.88% of the control. However, Mel-AF and Mel-AM did not show a significant difference in cell migration in NCI-H460 cells compared to the control. In this study, gemcitabine was also used as a positive control for proliferation inhibition [17]. The results showed that gemcitabine at 0.25 µg/mL directly inhibited the migration of all lung cancer cells, with % wound closure of 2.78 ± 0.38%, 0.40 ± 0.21%, and 1.18 ± 0.67%, for A549, NCI-H460 and NCI-H1975, respectively (Supplementary Figure S3).

**Figure 4:**
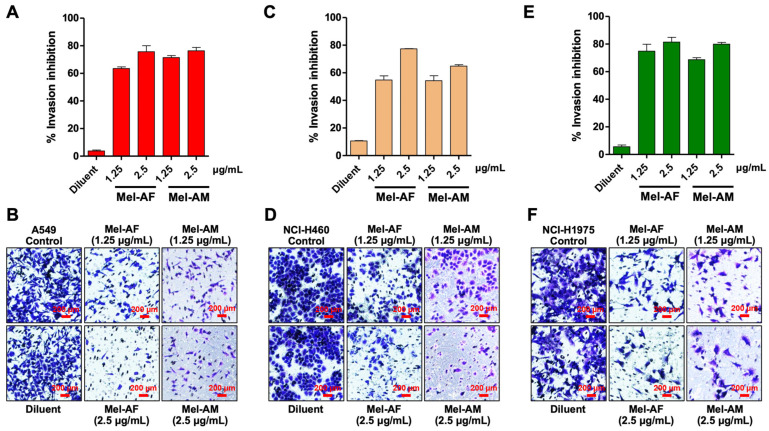
Effect of melittin peptides on cell invasion ability. Transwell invasion assay was performed to investigate the effect of Mel-AF and Mel-AM at concentrations of 1.25 and 2.5 µg/mL on different lung cancer cell lines: (**A**,**B**) A549, (**C**,**D**) NCI-H460, and (**E**,**F**) NCI-H1975. The percentages of invasion inhibition by Mel-AF and Mel-AM were calculated and compared with untreated cells (Control). Data are presented as mean ± SD values of at least three independent replicates.

Additionally, Mel-AF and Mel-AM at concentrations of 1.25 and 2.5 µg/mL demonstrated the ability to inhibit cell invasion in all lung cancer cells, with percentages of invasion inhibition ranging from 54.83 ± 5.16% to 81.46 ± 5.87% and 54.27 ± 7.44% to 79.92 ± 6.45% for Mel-AF and Mel-AM, respectively (Figure 4). Gemcitabine was also used as a positive control for proliferation inhibition [17]. The results showed that gemcitabine at 0.25 µg/mL inhibited the invasion of NCI-H460 and NCI-H1975 cells by more than 50%, with invasion inhibition percentages of 83.35 ± 3.83% and 73.31 ± 9.72%, respectively. However, in A549 cells, the invasion inhibition was 28.75 ± 9.85% (Supplementary Figure S4).

### 2.4. Expression of EGFR in Melittin-Treated Cells and Combination Assay of Melittin Peptides with Gefitinib

**Figure 5:**
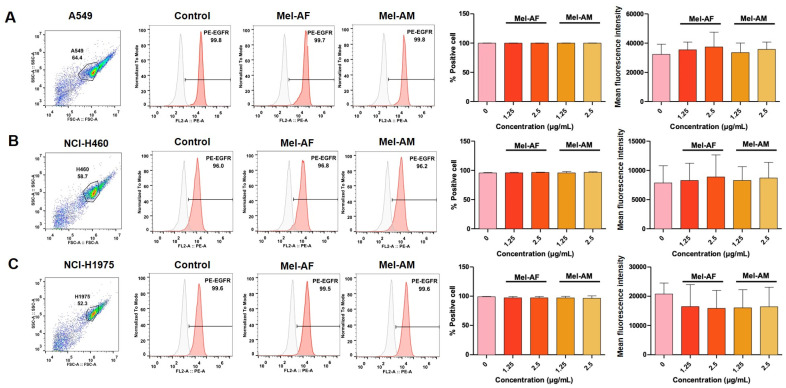
Expression of EGFR in melittin-treated cells. Mel-AF and Mel-AM at different concentrations (1.25 and 2.5 µg/mL) were used to treat lung cancer cell lines: (**A**) A549, (**B**) NCI-H460, and (**C**) NCI-H1975. Dot plots represent the flow cytometry gating strategy and histograms of EFGR positive cell gating (red) relative to isotype-matched control (grey). Percentages of positive cells and mean fluorescence intensity were determined using flow cytometry and compared between the melittin-treated and untreated cells. Data are presented as mean ± SD values of at least three independent replicates. Representative histograms from this experiment are presented in Supplementary Figure S5.

Epidermal Growth Factor Receptor (EGFR) proteins play several roles in cancer progression, including the promotion of cancer cell growth and resistance to chemotherapy [18]. In this work, we investigated the expression of EGFR in melittin-treated cells using flow cytometry. Interestingly, the results suggested that the EGFR expression of cells treated with Mel-AF and Mel-AM at 1.25 and 2.5 µg/mL was not significantly different compared to the untreated cells in all cell lines (Figure 5).

**Figure 6:**
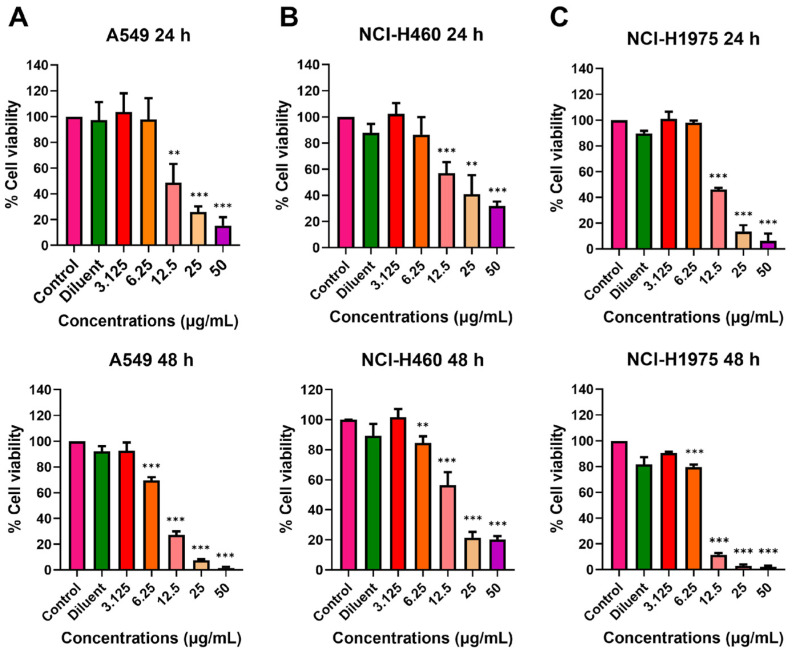
Cytotoxicity of gefitinib on lung cancer cells. Cytotoxicity of gefitinib was investigated on different lung cancer cell lines: (**A**) A549, (**B**) NCI-H460, and (**C**) NCI-H1975 after 24 h (top) and 48 h (bottom) of treatment. Data are presented as mean ± SD values of at least three independent replicates. Asterisk (*) indicates significance at *p* ≤ 0.01 (**) and *p* ≤ 0.001 (***).

A combination assay of melittin peptides with gefitinib, a chemotherapeutic drug that can inhibit EGFR [19], was then assumed to have synergistic effects against lung cancer cells. Prior to the combination assay, the cytotoxicity of gefitinib was evaluated in all cell lines used in this study. The results showed that, from the selected range of gefitinib between 0 and 50 µg/mL, the cytotoxic concentrations 50% (CC50s) of all cell lines were between 12.42 and 24.01 µg/mL at 24 h (Figure 6 (top)), by which NCI-H1975 was shown to be the most sensitive cell line (Figure 6 and Supplementary Table S2). According to these results, gefitinib at concentrations of 12.5, 25, and 50 µg/mL was selected for the combination assay with melittin peptides.

**Figure 7:**
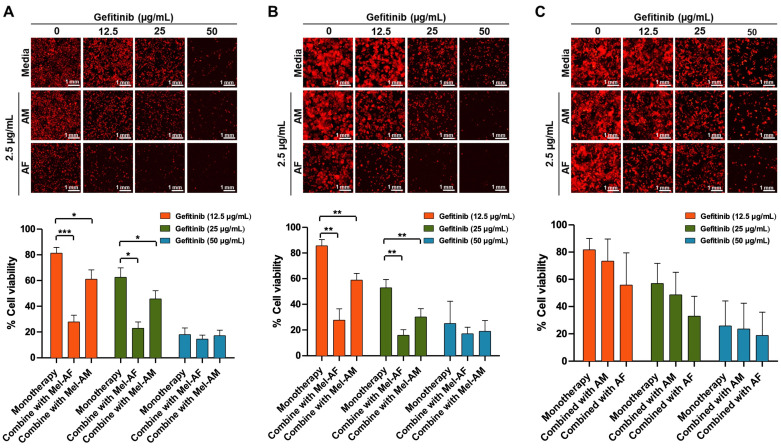
Combination of gefitinib and melittin peptides on lung cancer cells. Cell viability of (**A**) A549, (**B**) NCI-H460, and (**C**) NCI-H1975 cells after 24 h of combined treatment with gefitinib and melittin peptides. The number of cancer cells (expressing mCherry fluorescence protein) was investigated under a fluorescence inverted microscope. Data are presented as mean ± SD values of % cell viability relative to the untreated control from at least three independent replicates. Asterisk (*) indicates significance at *p* ≤ 0.05 (*), *p* ≤ 0.01 (**), and *p* ≤ 0.001 (***).

Mel-AF and Mel-AM at a concentration of 2.5 µg/mL were used in combination with gefitinib at 12.5, 25, and 50 µg/mL to treat all lung cancer cell lines. The results demonstrated the same trend in all cell lines, by which the combination of the gefitinib with either Mel-AF or Mel-AM showed enhanced inhibition effects compared with the use of melittin peptides or gefitinib alone (Figure 7). In the A549 cells, the percentage of cell viability was 81.28 ± 4.54 when treated solely with 12.5 µg/mL gefitinib. However, this value significantly decreased to 27.81 ± 5.22% and 61.05 ± 7.21% when the cells were subjected to combined treatment with Mel-AF and Mel-AM, respectively. Similarly, at a higher concentration of 25 µg/mL gefitinib, the initial cell viability percentage dropped to 65.53 ± 7.32% and further declined to 22.85 ± 4.95% and 45.74 ± 6.42% when administered in conjunction with Mel-AF and Mel-AM, respectively (Figure 7A). This pattern persisted in NCI-H460 cells, where the cell viability percentages decreased from 85.60 ± 4.84% to 27.66 ± 8.83% and 58.91 ± 5.21% upon treatment with 12.5 µg/mL gefitinib in combination with Mel-AF and Mel-AM, respectively. At a higher concentration of gefitinib (25 µg/mL), NCI-H460’s cell viability decreased from 52.85 ± 6.57% to 15.91 ± 4.29% and 30.11 ± 6.52% with Mel-AF and Mel-AM treatment, respectively (Figure 7B). Moreover, it was observed that Mel-AF showed a stronger inhibition effect compared with Mel-AM. In NCI-H1975 cells, the most tolerant cell line to melittins in this study (Figure 1), the reduction was not significant, yet a similar trend was observed (Figure 7C). Certainly, the use of a higher concentration of gefitinib at 50 µg/mL resulted in a more enhanced effect of gefitinib on the cells, but with this, no significant difference was observed between monotherapy with gefitinib and the combination assay with melittin peptides. However, it should be emphasized that the extra use of melittin peptides could reduce the necessity of using high doses of chemotherapeutic drugs, as, for example, the combination use of 2.5 µg/mL of Mel-AF with 12.5 µg/mL of gefitinib in NCI-H460 cells could reduce the % cell viability to 27.66 ± 8.83% compared to 25.06 ± 17.34% monotherapy with 50 µg/mL of gefitinib.

**Figure 8:**
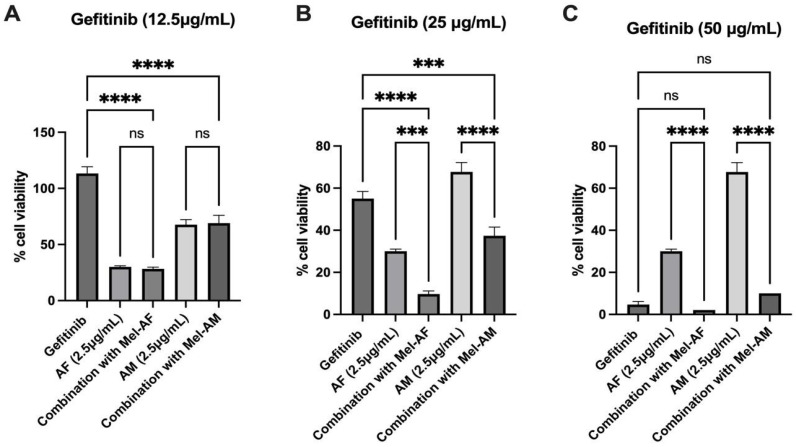
Combination of gefitinib and melittin peptides on NCI-H1650. Cell viability of NCI-H1650 after 24 h of combined treatment with different concentrations of gefitinib, (**A**) 12.5 µg/mL (**B**) 25 µg/mL (**C**) 50 µg/mL, and melittin peptides. Data are presented as mean ± SD values of % cell viability relative to the untreated control from at least three independent replicates. Asterisk (*) indicates significance at *p* ≤ 0.001 (***) and *p* ≤ 0.0001 (****).

Moreover, in the original cell lines selected for this study, only NCI-H1975 harbors EGFR mutations. Therefore, we included an additional lung cancer cell line, NCI-H1650, which also carries an EGFR mutation. Firstly, it was confirmed that melittin treatment did not alter the EGFR expression in any of the tested cell lines, including NCI-H1650 (Figure 5 and Supplementary Figure S6). Then, it was shown that the combination of gefitinib and melittin peptides led to a significantly greater reduction in cancer cell viability compared to treatment with either agent alone (Figure 8).

## 3. Discussion

Based on the Global Cancer Statistics for 2022, lung cancer stands as a leading cancer type in terms of incidence, representing 12.4% of total cases and 18.7% of cancer-related deaths worldwide [20]. Non-small cell lung cancer (NSCLC), presenting a higher incidence compared to small-cell lung cancer (SCLC), encompasses adenocarcinoma, squamous cell carcinoma, large cell carcinoma, and other subtypes identified through immunohistochemistry based on the World Health Organization (WHO) classification. In advanced stages of NSCLC, when surgery is not suitable, identifying other means of treatment becomes crucial. Thus, our primary objective was to comparatively investigate the effects of melittin peptides derived from two bee species, the dwarf honeybee (*A. florea*, Mel-AF) and the European honeybee (*A. mellifera*, Mel-AM), on A549, NCI-H460, and NCI-H1975 lung cancer cell lines.

The effects of melittin peptides on lung cancer cells have been investigated and primarily focused only on melittin derived from *A. mellifera*. In this work, it was clearly demonstrated that, at higher concentrations (5 and 10 µg/mL), both peptides exhibited significant or complete inhibition effects on all lung cancer cell lines (Figure 1). A previous study showed that melittin from *A. mellifera* suppressed the growth of NSCLC cell lines, A549 and H358, when a concentration range of 0–2.5 µg/mL was used [21]. Moreover, to demonstrate their selectivity, Vero cells were used as a non-cancerous cell line in this study. Additionally, one study has investigated the effects of melittin from *A. mellifera* on BEAS-2B cells, an immortalized normal lung epithelial cell line, and observed only a slight effect, even at the highest tested concentration of 4 µg/mL [12].

Noticeably, Mel-AF was shown to have more renounced effects in all experiments compared to Mel-AM, suggesting its more invasive properties in cancer cell inhibition. A previous study has also shown varying activities between Mel-AF and Mel-AM, where Mel-AF emerged as a more toxic candidate against human malignant melanoma (A375) cells [22]. The underlying mechanisms behind this difference may involve the cationic properties of the peptides, which attract cancer cells that tend to be more electronegative than normal cells [23]. Thus, it stands to reason that if these peptides are more positively charged, they should exhibit a greater toxicity toward cancer cells. Moreover, the hydrophobic interaction between peptides and cancer cells is also considered to impact selectivity, by which a higher hydrophobicity results in stronger inhibitory activity with a necrotic-like membrane disruption mechanism [24]. The helicity of peptides has also been systematically characterized, showing that a lower helicity can reduce toxicity against HeLa cancer cells [25]. A melittin peptide from *A. mellifera* was previously modified by replacing two valine residues in the peptide chain to increase its charge and helicity, resulting in a decreased IC50 against hepatoma cells [26]. In our case, the differences between the Mel-AF and Mel-AM sequences were found to be 5 out of the 27 amino acids (Supplementary Figure S7A). However, these different amino acid residues do not affect the net charge (equal net charge at pH 7.0 of 5.00) and hydrophobicity (equal 70% hydrophobic amino acids) of the peptides (Supplementary Figure S7B,C). Moreover, only the crystal structure of melittin from *A. mellifera* was reported (PDB: 2MW6), so PEP-FOLD3, an online tool for peptide structure modeling (https://bioserv.rpbs.univ-paris-diderot.fr/services/PEP-FOLD3/, accessed on 17 March 2024), was used to predict the helicity of Mel-AF using Mel-AM’s structure as a reference. It was found that the helicity of these peptides was similar (Supplementary Figure S7D,E). However, when the sequences and structures were examined closely, referencing proline as a helix-breaking amino acid, the peptides could be divided into two fragments. The left fragment (Supplementary Figure S7A) of Mel-AF was shown to contain isoleucine at position 5, whereas it was valine for Mel-AM. Since isoleucine has a longer alkyl chain compared with valine, this could result in a slightly higher hydrophobicity on this side of the peptide. It is possible that this hydrophobicity enables the peptide to disrupt or traverse the cell membrane more rapidly by integrating this side of the peptide into the lipid bilayers; however, a comprehensive investigation is required to confirm this. Overall, it suggests that rational design can enhance the activity and selectivity of the melittin peptide. For instance, AC-P19M was de novo designed and modified by replacing amino acids with those with a higher cationicity and hydrophobicity, such as arginine (R) and lysine (K). This modification led to an increased selectivity toward A549 and NCI-H460 cells [27]. Moreover, lung cancer cells are known to exhibit a high expression of EGFR, which is located on the cell membrane. Molecular docking analyses of EGFR with Mel-AF and Mel-AM revealed that both peptides bind to EGFR near the EGF docking site (Supplementary Figure S8). This suggests that Mel-AF and Mel-AM may competitively bind to EGFR, potentially inhibiting EGF interaction and leading to reduced cell proliferation.

Other studies have shown that melittin peptides were able to induce the intrinsic apoptosis pathway in gastric cancer (SGC-7901 cells), colorectal cancer (HCT-116 and SW-480 cells), and ovarian cancer (SKOV3 and PA-1 cells). Although melittin peptides have not been reported to induce apoptosis through the intrinsic mitochondrial pathway in lung cancer cells, other pathways have been reported. Previously, melittin was shown to induce the apoptosis of NSCLC cells, NCI-H441, via the inhibition of miR-183 [12]. Caspase 2 was reported to be the direct target of miR-183, by which the inhibition of miR-183 expression resulted in an increased CASP2 expression to inhibit the proliferation and tumor growth of NCI-H441 cells [12]. Moreover, the downregulation of the TGF-β-mediated ERK signal pathway was observed in melittin-treated A549 and H358 lung cancer cells, leading to cell apoptosis [21]. An attempt to explain the apoptosis pathway in A549 and NCI-H460 lung cancer cells has been demonstrated, elucidating that the apoptotic cell death caused by bee venom was through the enhancement of DR3 expression and inhibition of the NF-κB pathway [13]. Moreover, a previous study utilizing the same Mel-AF and Mel-AM as in this study indicated that, in human malignant melanoma (A375) cells, the translocation of cytochrome C from the mitochondria to the cytosol was also observed, which is consistent with the activation of the intrinsic mitochondrial pathway [22]. However, it is important to consider that the observed effects of melittin peptides on cells may be influenced by alterations in several signaling pathways.

Having an ability to inhibit cell migration and invasion are promising characteristics of therapeutic agents against cancer cells. Our results suggested that Mel-AF and Mel-AM significantly suppressed the migration of A549 and NCI-H1975 and the invasion of all tested lung cancer cells at the concentration of 2.5 µg/mL. This is in agreement with a previous study, where the migration and invasion abilities of A549 and H358 cells were markedly inhibited by 2.0 µg/mL of melittin [21]. In another study, similar findings were demonstrated and indicated that the inhibition was EGF-induced [11]. Moreover, a melittin peptide significantly reduced the expression level of miR-183, leading to a decrease in the invasion and migration abilities of NCI-H441, another NSCLC cell line [12]. Certainly, this inhibitory effect on cell proliferation could contribute to the observed reduction in migration and invasion. To further clarify this distinction, we assessed migration and invasion following treatment with the proliferation inhibitor, gemcitabine (0.25 µg/mL). The results showed that gemcitabine significantly reduced both migration (Supplementary Figure S3) and invasion (Supplementary Figure S4) across all tested lung cancer cell lines, indicating that proliferation inhibition plays a role in these processes. However, Mel-AF and Mel-AM exhibited similar inhibitory effects on migration and invasion as gemcitabine, suggesting that their impact may, at least in part, be linked to proliferation suppression.

Previously, a melittin peptide from *A. mellifera* was demonstrated to suppress the activation of EGFR in breast cancer cells [28]. However, these findings are in contrast to a study conducted by Shin et al. (2013), where it was revealed that melittin had no effect on the phosphorylation levels of EGFR, p38, JNK, and Akt [29]. Moreover, a previous work on skin cancer cells using the same Mel-AF peptide as this work also suggested that this peptide could diminish EGFR activity by suppressing its expression [22]. In our study, we did not observe significant difference in EGFR expression between Mel-AF or Mel-AM treated and untreated cells. This could be due to several factors. Firstly, the 24 h treatment duration utilized in our study might not have been optimal. The aforementioned study in skin cancer cells that noted a significant reduction in EGFR expression employed a shorter 2 h treatment with Mel-AF [22]. Secondly, as a reduction in EGFR expression as a result of melittin treatment has never been reported in lung cancer cells, this could be because EGFR in lung cancer cells is highly expressed [30], potentially leading to a reduced sensitivity to melittin-induced effects.

The combination of natural products helps to alleviate the side effects of chemotherapeutic drugs, as enhanced effects can be observed with relatively lower doses [31]. Melittin peptides have been shown to be effective in combination therapies. For example, a previous study demonstrated the use of a melittin peptide in combination with Sorafenib against HepG2 cells. Such a combination resulted in the upregulation of tumor suppressor genes and the downregulation of oncogenes [15]. Gefitinib, a tyrosine kinase inhibitor (TKI) that targets EGFR, is a first-line chemotherapeutic drug for treating NSCLC, particularly in patients with EGFR mutations [32,33]. It has also been demonstrated that gefitinib monotherapy is effective in patients with advanced NSCLC [34]. However, acquired resistance is common in patients with the secondary mutation, T790M, in EGFR. This T790M mutation arises from a C>T substitution at nucleotide 2369 in exon 20 of the EGFR gene, resulting in the replacement of threonine with methionine at position 790 (T790M). This mutation has been reported in approximately 50% of patients with acquired resistance to first-generation EGFR tyrosine kinase inhibitors (TKIs), such as gefitinib and erlotinib, in NSCLC patients with activating EGFR mutations [35]. Threonine 790, known as the “gatekeeper residue” in EGFR, is positioned at the entrance of a hydrophobic pocket within the ATP-binding cleft, playing a crucial role in determining kinase inhibitor specificity. Substituting threonine with the bulkier methionine (T790M) induces resistance by creating steric hindrance, which disrupts gefitinib binding [36,37]. Additionally, this mutation restores EGFR’s ATP affinity to wild-type levels, further reducing the effectiveness of gefitinib [36]. Consequently, EGFR signaling remains active, promoting cancer cell survival and proliferation.

In our study, the combination of melittin peptides and gefitinib showed enhanced therapeutic effects compared with separated treatments. However, the results from NCI-H1975, which carries the L858R and T790M mutations, showed the lowest efficiency. The L858R mutation in NCI-H1975 typically responds well to gefitinib, while the T790M mutation leads to a reduced sensitivity [33,38]. To further investigate the efficiency of melittin in EGFR-mutant cells, we used NCI-H1650 carrying the T790M EGFR mutation as an additional model. Consistent with our observations in other cell lines, melittin treatment did not alter the EGFR surface expression levels (Figure 6). As expected, NCI-H1650 cells exhibited resistance to gefitinib (Supplementary Figure S9). Notably, the combination of gefitinib and melittin significantly increased cancer cell death compared to single-agent treatment (Figure 8). These findings further support the potential of melittin in sensitizing cancer cells to chemotherapeutic drugs, particularly in EGFR-mutant lung cancer. In contrast, the A549 and NCI-H460 cell lines used in this study possess wild-type EGFR, with no EGFR mutations [39]. It should be noted that A549 and NCI-H460 cell lines have been reported as EGFR-independent cells, which could have caused the resistance to gefitinib in these cells. Interestingly, from our results, a combination of Mel-AF with low-dose gefitinib (12.5 and 25 µg/mL) resulted in enhanced inhibitory effects compared to gefitinib alone at a higher concentration (50 µg/mL). These results are in line with a previous study, TRAIL, where the combination use of bee venom with different therapeutic drugs, docetaxel or cisplatin, further inhibited A549 and NCI-H460 cell growth [13]. Previously, the combination use of a melittin peptide with anti-cancer antibodies (Bevacizumab and Cetuximab) has been reported in A549 and HepG2 cancer cells, and synergistic effects were also observed [40]. This emphasizes the efficiency of using a combination treatment of melittin peptides and gefitinib in patients with wild-type EGFR but EGFR-independent cancers.

Altogether, this work demonstrated that the melittin peptides from both bee species exhibit potential for use as chemotherapeutic agents, either alone or in combination with other drugs. Apart from its aforementioned biological properties, the melittin from *A. mellifera* has been shown to inhibit HIF-1α, which, in turn, modulates the tumor microenvironment, including processes such as angiogenesis [41]. Among these, Mel-AF emerged as the candidate with more pronounced effects. The enhanced efficacy observed when combined with gefitinib allows for a reduced dosage of the drug, thereby minimizing its side effects. Recent studies have demonstrated delivery systems that enhance the specificity of melittins toward cancer cells, thereby increasing their toxicity and therapeutic efficacy [42,43]. Moreover, upon clinical use, upscaling the production of Mel-AF and Mel-AM is a future avenue for development. Regarding synthesis, chemical or recombinant methods can be employed to produce melittin peptides on a large scale. These methods ensure consistency, scalability, and cost-effectiveness compared to direct extraction from bees. Several studies have reported the successful recombinant production of melittin using *Escherichia coli* as a host. This approach leverages the efficiency and scalability of bacterial expression systems, enabling high-yield production [44,45,46].

## 4. Materials and Methods

### 4.1. Synthetic Melittin Peptides and Cell Cultures

Two melittin peptides derived from 2 different species of bees, *Apis florea* (Mel-AF) and *Apis mellifera* (Mel-AM), were purchased from Ward medic (Bangkok, Thailand) and synthesized by GenScript (Jiangsu, China) with a purity of ≥98%, as qualified via both mass spectrometry (MS) and high-performance liquid chromatography (HPLC) analyses. The sequences of each peptide were as follows:

Mel-AF—GIGAILKVLATGLPTLISWIKNKRKQG and

Mel-AM—GIGAVLKVLTTGLPALISWIKRKRQQG.

The synthesized peptides were diluted in sterile distilled water prior to use.

The following three different NSCLC cell lines were used: A549, NCI-H460, and NCI-H1975. These cell lines were obtained from American Type Culture Collection (CCL-185, CRL-5801, CRL-5908; American Type Culture Collection (ATCC), Manassas, VA, USA). All cell lines were cultured in RPMI media (Gibco, Thermo Fisher Scientific, Waltham, MA, USA) with 10% FBS and 1% penicillin/streptomycin. NCI-H1650 cells were also used as an additional EGFR mutant cell line cultured in the same medium. Monkey kidney epithelial Vero cells (CCL-81; American Type Culture Collection (ATCC), Manassas, VA, USA) were also used in this study as a non-cancerous control. They were cultured in a minimal essential medium (MEM) supplemented with 10% (*v*/*v*) fetal bovine serum (FBS) (Gibco; Thermo Fisher Scientific, Waltham, MA, USA) and 1% penicillin/streptomycin (Gibco, Thermo Fisher Scientific, Waltham, MA, USA). All cells were incubated in a 5% CO_2_ incubator (ESCO MEDICAL, Egå, Denmark) at 37 °C.

### 4.2. Cell Viability Assay

The effect of melittin peptides on cell death was determined. In brief, all cell lines—A549, NCI-H460, and NCI-H1975 and also the control, Vero—were plated with 10,000 cells/well in 96-well plates and incubated for 24 h at 37 °C in a 5% CO_2_ incubator prior to the treatment with different concentrations (0.3125, 0.625, 1.25, 2.5, 5, and 10 µg/mL) of Mel-AF and Mel-AM. After the treatment, all cell lines were incubated for 24 and 48 h. To assess the cell viability, PrestoBLUE™ cell viability reagent (Thermo Fisher Scientific, Waltham, MA, USA) was used according to the manufacturer’s instructions. The absorbance at 570 nm and 595 nm was measured using a microplate reader (EZ Read 2000, Biochrom, Cambridge, UK) and the values were calculated for % cell viability compared to that of the non-treated control. For MTT assay, 2 mg/mL of MTT solution (Bio Basic Inc., Amherst, NY, USA) was then added to each well, and the plates were incubated for 3 h. Blue formazan crystals formed during the assay were dissolved with dimethyl sulfoxide (DMSO), and the absorbance was measured at 540 nm and 630 nm using a microplate reader (EZ Read 2000, Biochrom, Cambridge, UK). The percentage of cell viability was calculated by comparing the absorbance values of treated samples to those of the untreated cell control.

Similarly, the cytotoxicity of gefitinib (Sigma-Aldrich, CAS 184475-35-2, Buchs, Switzerland) against lung cancer cell lines was investigated prior to its use in combination assays. Three lung cancer cell lines were plated with 15,000 cells/well. The cells were incubated for 24 h and treated with gefitinib at concentrations of 3.12, 6.25, 12.5, 25, and 50 µg/mL. The cells were incubated for 24 and 48 h and their viability was evaluated using PrestoBLUE™ cell viability reagent.

### 4.3. Flow Cytometry

Flow cytometry was used to assess cell death and surface epidermal growth factor receptor (EGFR) expression levels. For cell death assessment, A549, NCI-H460, and NCI-H1975 cells were plated in 48-well plates (2 × 10^4^ cells/ well) 24 h before the experiment, then treated with melittin peptides at concentrations of 1.25, 2.5, and 5 µg/mL for 4 h. The cells were harvested and stained with AnnexinV conjugated APC and propidium iodide (PI) (ImmunoTools, Friesoythe, Germany). The stained cells were analyzed by flow cytometry using CytoFlex LX (Backman Coulter, Brea, CA, USA). The data were analyzed using CytExpert Software v2.6 (Backman Coulter, Brea, CA, USA) to calculate the percentages of early apoptosis (AnnexinV+ only), late apoptosis (AnnexinV+/PI+), or necrosis (PI+ only) compared to that of the non-treated control.

The effect of melittin peptides on the expression level of surface EGFR was also determined. Briefly, A549, NCI-H460, and NCI-H1975 cells were plated in 48-well plates (2 × 10^4^ cells/ well) 24 h before the experiment. The cells were treated with melittin peptides at the concentrations of 1.25 and 2.5 µg/mL for 24 h. The cells were collected, followed by staining with a monoclonal antibody specific to EGFR (BioLegend, San Diego, CA, USA), and then analyzed using CytoFlex LX. The data were analyzed using CytExpert Software v2.6 to determine the percentage of positive cells compared to that of the non-treated control.

### 4.4. Western Blot Analysis

Western blot analysis was used to investigate the apoptosis pathways. A549, NCI-H460, and NCI-H1975 cells were plated with 1 × 10^5^ cells/well in 24-well plates and incubated at 37 °C in a 5% CO_2_ incubator for 24 h before the experiment. The cells were treated with Mel-AF and Mel-AM (at concentrations of 1.25, 2.5, and 5 µg/mL). The plate was then incubated for 2 h at 37 °C in a 5% CO_2_ incubator. The supernatants were removed and the whole cell lysate was collected using 100 µL of 1X reducing buffer (1X RSB). The sample was heated at 95 °C for 10 min before it was loaded into 12% Sodium Dodecyl Sulfate-Polyacrylamide Gel Electrophoresis (SDS-PAGE) and transferred to a nitrocellulose membrane. After that, membranes were blocked with 5% skim milk in 0.1% TBST for 1 h at room temperature. Next, the membranes were probed with primary antibody (1:1000 dilution in 5% skim milk in 0.1% TBST), including procaspase 9, procaspase 8, procaspase 3, and Bcl-2 (1:500 dilution), and incubated at 4 °C overnight. Membranes were washed with 0.1% TBST for 5 min 3 times and incubated for 1 h at room temperature with specific anti-rabbit or anti-mouse secondary antibodies (1:1000 dilution in 5% skim milk in 0.1% TBST). The membranes were washed with 0.1% TBST 3 times (5 min each) before detection using a chemiluminescence agent (Thermo scientific, Rockford, IL, USA) on an ImageQuant LAS 500 chemiluminescence CCD camera (GE Healthcare Bio-Sciences AB, Danderyd, Sweden). The results were analyzed by ImageJ analysis software, ImageJ bundled with 64-bit Java 8. The protein levels were normalized with GADPH protein.

### 4.5. Scratch Wound and Transwell Invasion Assays

The effect of Mel-AF and Mel-AM on cell migration ability was determined using scratch wound assay. Cells were plated at a density of 2 × 10^5^ cells/mL in 24-well plates and incubated at 37 °C in a 5% CO_2_ incubator for 24 h. The cells were then scratched with a 10 µL pipette tip to create a wound. Afterward, the cells were washed with PBS to remove any detached cells. Media containing Mel-AF and Mel-AM were added, and the cells were incubated at 37 °C in a 5% CO_2_ incubator for 24 h. Migration images were captured at reference points, and the gap area distance was measured using the Image J program before calculating the percentage of wound closure compared to untreated cells [47]. The percentage of wound closure was calculated and compared to that of non-treated control, as follows.

(1) $$\%\;\mathrm{Wound}\;\mathrm{closure}=(\frac{{\mathrm{gap}\;\mathrm{distance}}_{t0}-{\mathrm{gap}\;\mathrm{distance}}_{t24/t48}}{{\mathrm{gap}\;\mathrm{distance}}_{t0}})\times 100$$

The effect of Mel-AF and Mel-AM on cell invasion ability was determined using Matrigel (Corning, New York, NY, USA)-coated transwell invasion assay. This specialized transwell system ensures that only invading cells can pass through the Matrigel layer [48]. The upper chamber of a 24-well transwell inserted with an 8 µm pore size polyethylene terephthalate membrane (NEST, Wuxi, China) was coated with 0.4% Matrigel (Corning, New York, NY, USA) and incubated at 37 °C in a 5% CO_2_ incubator for 6 h. The Matrigel solution was then removed and 100 µL of cells (2 × 10^5^ cells/mL) was added to the upper chamber. Then, serum-free media containing Mel-AF and Mel-AM was added to the upper chamber and 600 µL of complete media was added to the lower chamber. After incubating for 24 h, the non-invaded cells in the upper compartment were gently removed with cotton swabs. The upper chamber containing the cells that passed through the Matrigel and were located on the underside of the membrane was washed with PBS twice. The cells were fixed with 3.7% formaldehyde for 5 min and permeabilized with methanol for 20 min. Subsequently, the cells were stained with 0.5% crystal violet for 30 min. After washing and air drying, cells were counted using an inverted microscope and the percentage of invasion inhibition was calculated compared to that of the non-treated control [47]. The percentage of invasion inhibition was calculated and compared to that of the non-treated control, as follows.

(2) $$\%\;\mathrm{Invasion}\;\mathrm{inhibition}=(\frac{C_{\mathrm{control}}-C_{\mathrm{treated}}}{C_{\mathrm{control}}})\times 100$$

where C_control_ is the number of invaded cells in the non-treated control and C_treated_ is the number of invaded cells in the treated group.

### 4.6. Combination Assay of Melittin Peptides with Gefitinib

A combination assay of melittin peptides and gefitinib was performed with all three lung cell lines used in this work. The cells were plated in a 96-well plate in the same manner as the aforementioned cytotoxicity test in a 96-well plate. The cells were treated with 2.5 µg/mL of melittin peptides in combination with 12.5, 25, and 50 µg/mL of gefitinib for 24 h. After the incubation, viable cells attached on the plate were detected using a fluorescence inverted microscope (Nikon ECLIPSE Ts2R, Tokyo, Japan) and crystal violet staining. To obtain quantitative data, the cells that were stained with crystal violet were resolved with 50% (*v*/*v*) ethanol and the absorbance of OD_595_ was measured using a microplate reader (EZ Read 2000, Biochrom, Cambridge, UK). The percentage of cell viability was calculated relative to the non-treated control.

### 4.7. Statistical Analysis

All results presented in this work were averages from at least three independent experiments. The statistical analyses were performed using GraphPad Prism version 9 software (GraphPad Software, Inc., San Diego, CA, USA). A *p*-value of ≤0.05 was considered significant.

## 5. Conclusions

In summary, melittin peptides, Mel-AF and Mel-AM, showed inhibitory effects toward all NSCLC cell lines used in this work (A549, NCI-H460, and NCI-H1975). The effect was rapid, as apoptosis was observed after only 4 h of the treatment through an intrinsic mitochondrial pathway. Both Mel-AF and Mel-AM could also inhibit cancer cell migration and invasion abilities; however, they did not impact EGFR expression. The combination uses of Mel-AF and Mel-AM with gefitinib, a chemotherapeutic drug targeting EGFR, showed an enhanced impact in comparison with the use of either Mel-AF, Mel-AM, or gefitinib separately. Altogether, this work demonstrated that melittin peptides from *A. florea* (Mel-AF) and *A. mellifera* (Mel-AM) showed potential as cytotoxic agents against NSCLC cells, with Mel-AF having more significant effects compared with Mel-AM, and the combination of these peptides with gefitinib resulted in more pronounced effects, potentiating their practical use in cancer treatment. The future development of nanotechnology-based delivery systems could enable the targeted and specific delivery of melittin peptides, enhancing their practicality for cancer treatment. Nevertheless, comprehensive in vivo studies and clinical trials are necessary to confirm their effectiveness, half-life, and safety, as well as to better understand the efficacy and cytotoxicity of the melittin in vivo.

## Data Availability

The original contributions presented in this study are included in the article and Supplementary Materials. Further inquiries can be directed to the corresponding author.

## Supplementary Materials

The following supporting information can be downloaded at: https://www.mdpi.com/article/10.3390/ijms26062498/s1.
