# Supplementary material for: A Comparative Study of Melittins from Apis florea and Apis mellifera as Cytotoxic Agents Against Non-Small Cell Lung Cancer (NSCLC) Cells and Their Combination with Gefitinib
Source: Int J Mol Sci. 2025 Mar 11;26(6):2498. doi: 10.3390/ijms26062498 (PMC11941996; doi:10.3390/ijms26062498)
Supplement: Supplementary file 1 [file ijms-26-02498-s001.zip › ijms-3433732-supplementary.pdf]

Supplementary information

Table S1. Cytotoxic concentration 50% (CC50) values of melittin peptides on lung cancer cells

Cells	CC50 values (µg/mL)			
	Mel-AF		Mel-AM	
	24 h	48 h	24 h	48 h
A549	2.55	3.52	4.32	5.27
NCI-H460	2.61	4.99	5.10	7.60
NCI-H1975	5.06	7.05	8.48	>10

Table S2. Cytotoxic concentration 50% (CC50) values of gefitinib on lung cancer cells

Cells	CC50 values of gefitinib (µg/mL)	
	24 h	48 h
A549	15.70	8.598
NCI-H460	24.01	14.75
NCI-H1975	12.42	8.255

Figure S1. Cytotoxicity of melittin peptides on Vero cells.

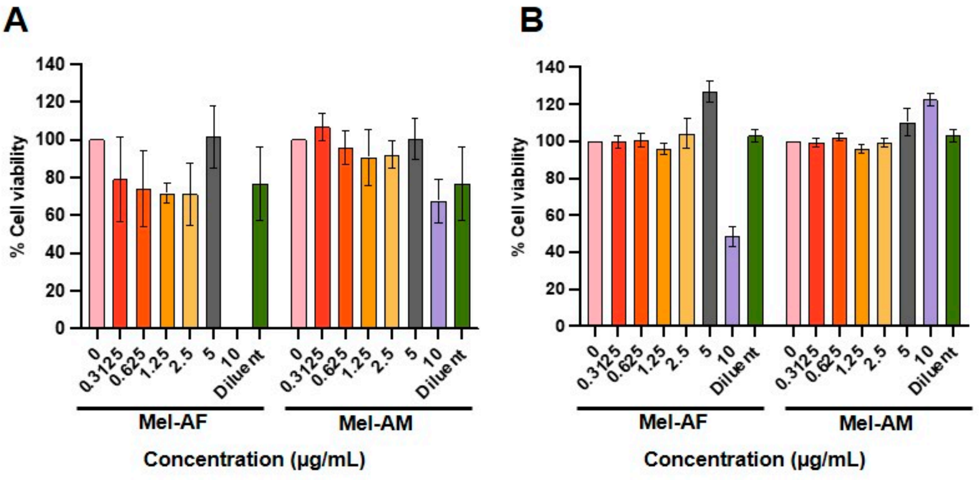

Vero cells were treated with Mel-AF and Mel-AM for (**A**) 24 h and (**B**) 48 h. Cell viability was assessed using PrestoBlue™ assay.

Figure S2. Western blot analysis to determine apoptosis pathway.

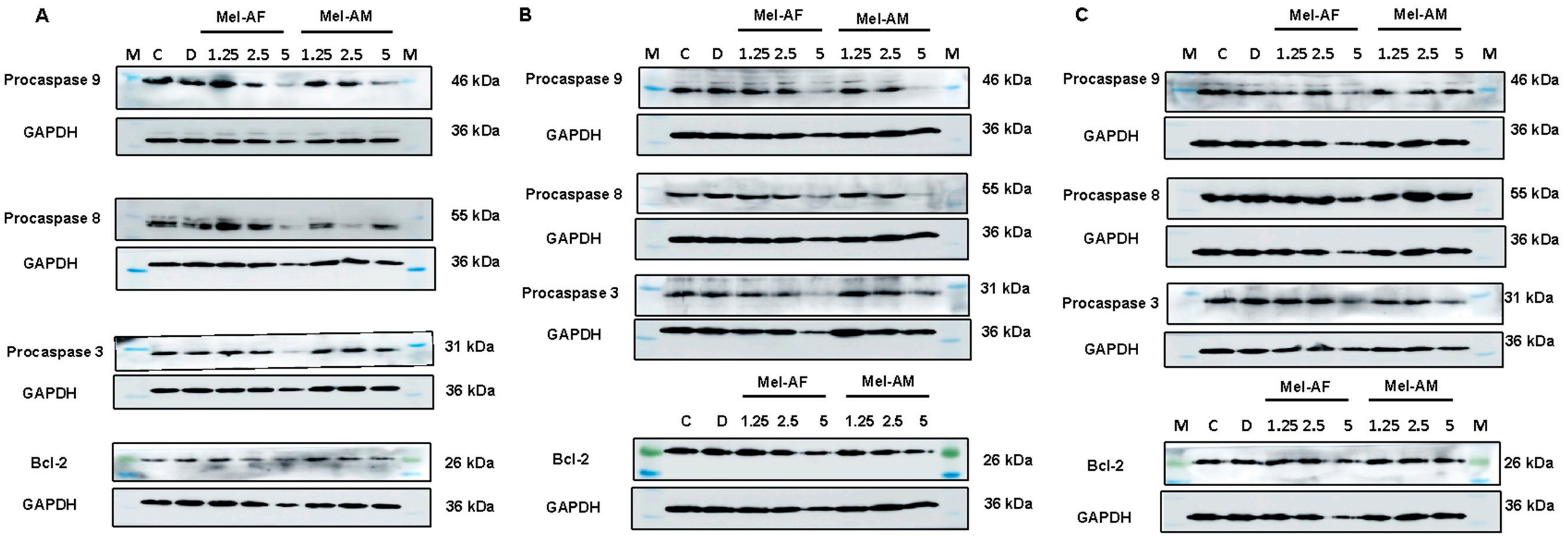

Expression of apoptosis proteins: procaspase 9, procaspase 8, procaspase 3 and anti-apoptosis protein: Bcl-2 after 2 h of melittin treatments in (**A**) A549, (**B**) NCI-H460 and (**C**) NCI-H1975 cells.

Figure S3. Effect of gemcitabine on cell migration ability.

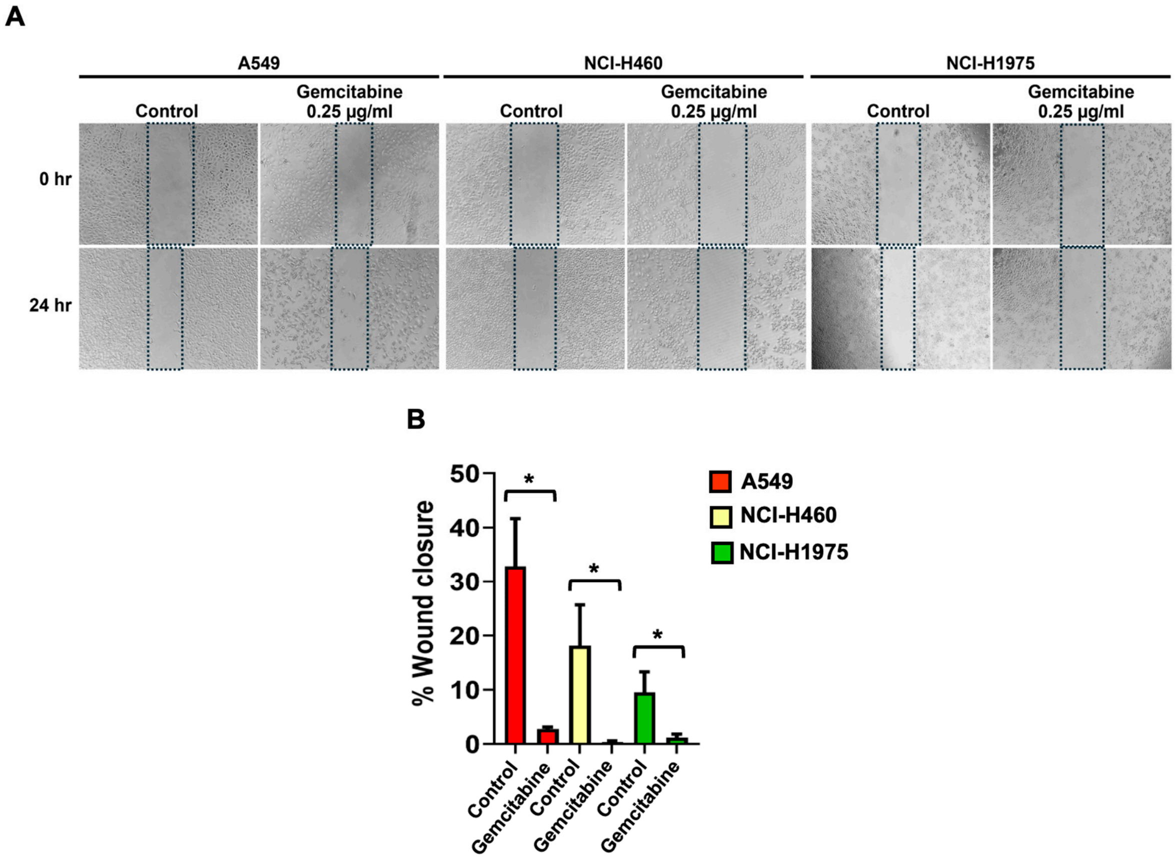

Suppression of cell migration ability by gemcitabine at 0.25 µg/mL on different lung cancer cell lines: A549, NCI-H460, and NCI-H1975. The cell migration was investigated using a scratch wound assay when compared with untreated cells (Control). (**A**) Images depicting wounds at 0 and 24 h after treatment with gemcitabine are shown and (**B**) the % wound closure is also presented. Data are presented as mean ± SD values of at least three independent replicates. Asterisk (*) indicates significance at $p \leq 0.05$ (*).

Figure S4. Effect of gemcitabine on cell invasion ability.

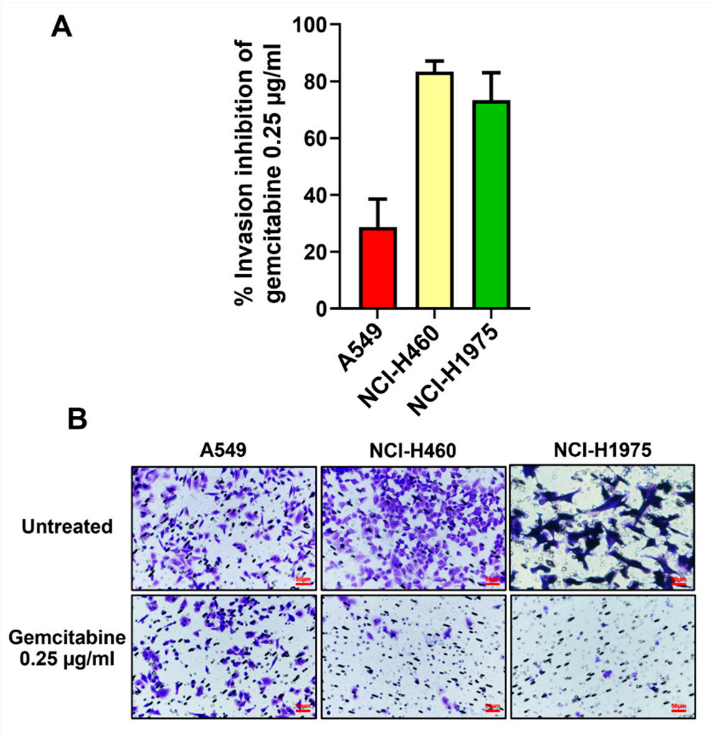

Transwell invasion assay was performed to investigate the effect of gemcitabine at a concentration of 0.25 µg/mL on different lung cancer cell lines: A549, NCI-H460, and NCI-H1975. (**A**) The percentages of invasion inhibition were calculated and compared with untreated cells (Control) and (**B**) the representative photos are also presented. Data are presented as mean ± SD values of at least three independent replicates.

Figure S5. Histograms representing EGFR expression via flow cytometric analysis.

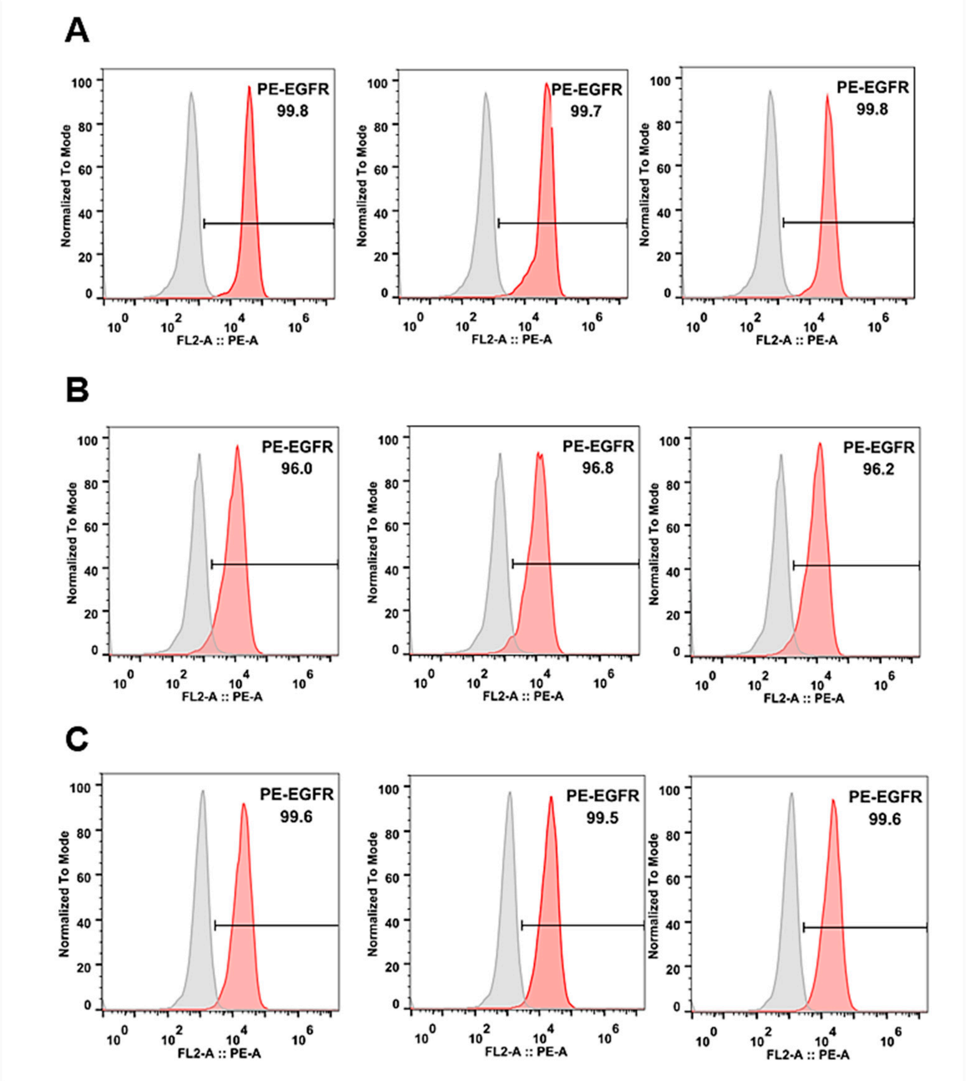

EGFR expression was analyzed using flow cytometry. All lung cancer cell lines: (**A**) A549 (**B**) NCI-H460, and (**C**) NCI-H1975, were treated with 5 µg/mL of Mel-AF (middle) and Mel-AM (right). Untreated cells are also presented (left). The histograms represent the EGFR expressing cell population (red) compared to isotype control (grey).

Figure S6. EGFR expression in NCI-H1650 cells and alteration after melittin treatment.

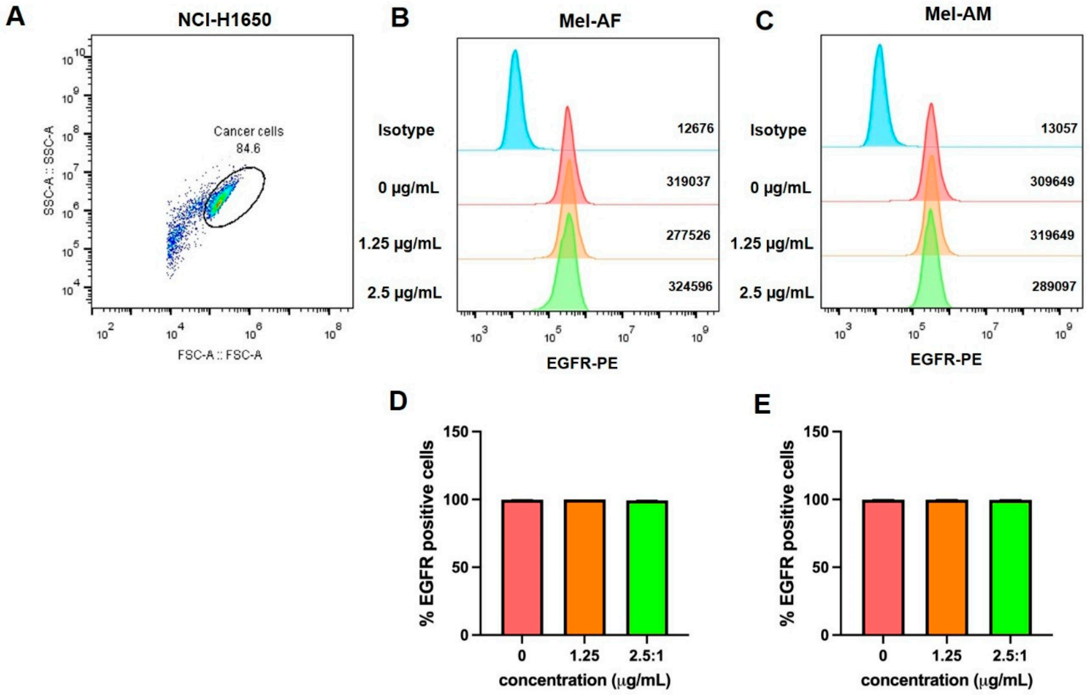

NCI-H1650 was used in this experiment to investigate the effect of melittin on EGFR expression. Mel-AF and Mel-AM at different concentrations (1.25 and 2.5 μg/mL) were used to treat the lung cancer cells. (**A**) Dot plots represent the flow cytometry gating strategy and histograms of EFGR positive cell gating (red) relative to isotype-matched control (blue). Representative histograms for (**B**) Mel-AF and (**C**) Mel-AM from this experiment are presented. (**D,E**) Percentages of EGFR positive cells were determined using flow cytometry and compared between the melittin-treated and untreated cells. Data are presented as mean ± SD values of at least three independent replicates.

Figure S7. Amino acid alignment between Mel-AF and Mel-AM and their characterization.

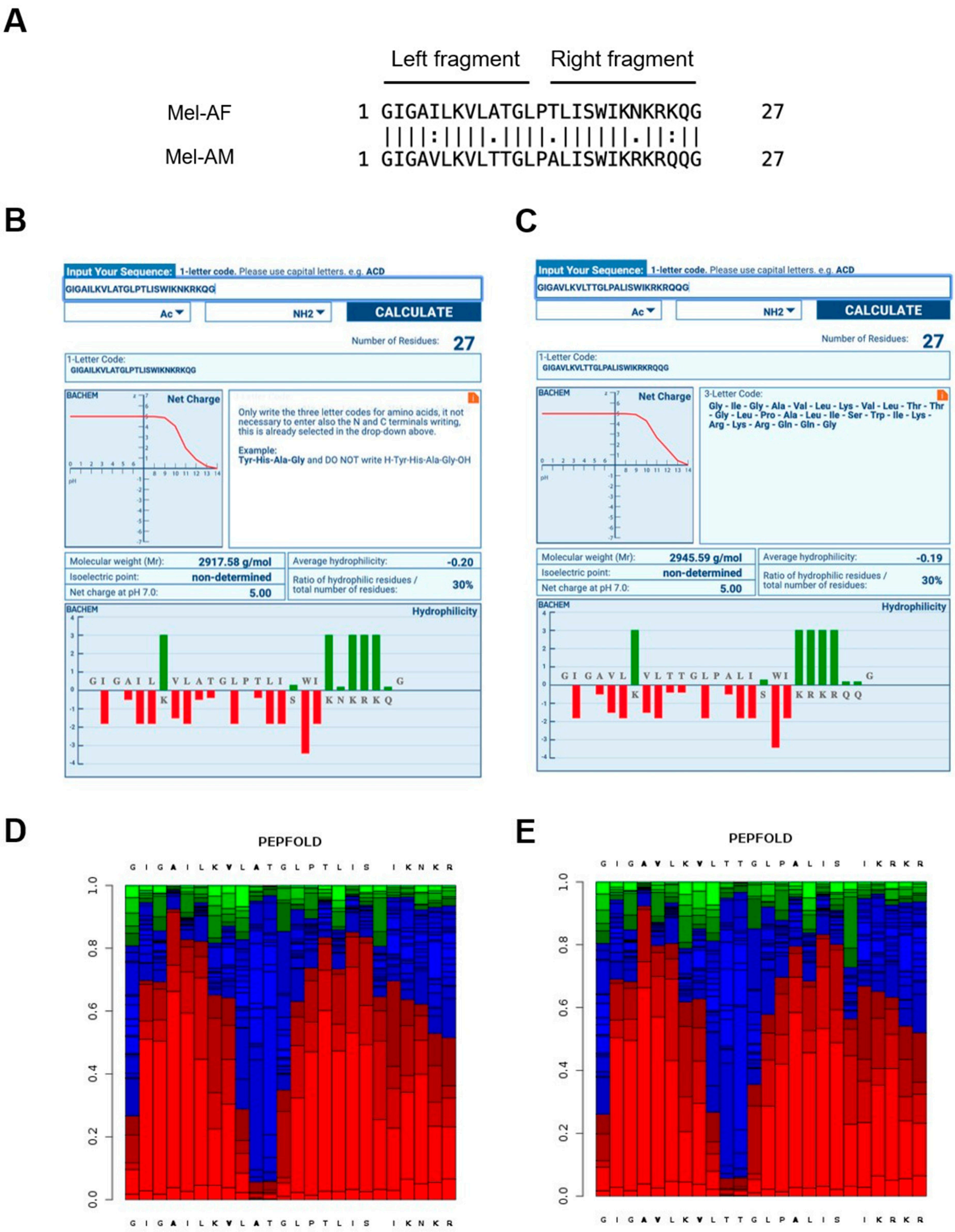

(**A**) Amino acid alignment between Mel-AF and Mel-AM with (**B,C**) their characteristics from an online peptide calculator (https://www.bachem.com/knowledge-center/peptide-calculator/). PEP-FOLD, an online peptide modelling tool, (https://bioserv.rpbs.univ-paris-diderot.fr/services/PEP-FOLD/) was also used to predict the structure and helicity of (**D**) Mel-AF and (**E**) Mel-AM. The profile is presented using the following color code: red: helical, green: extended, blue: coil.

Figure S8. Molecular docking analysis of epidermal growth factor receptor (EGFR) and melittin peptides.

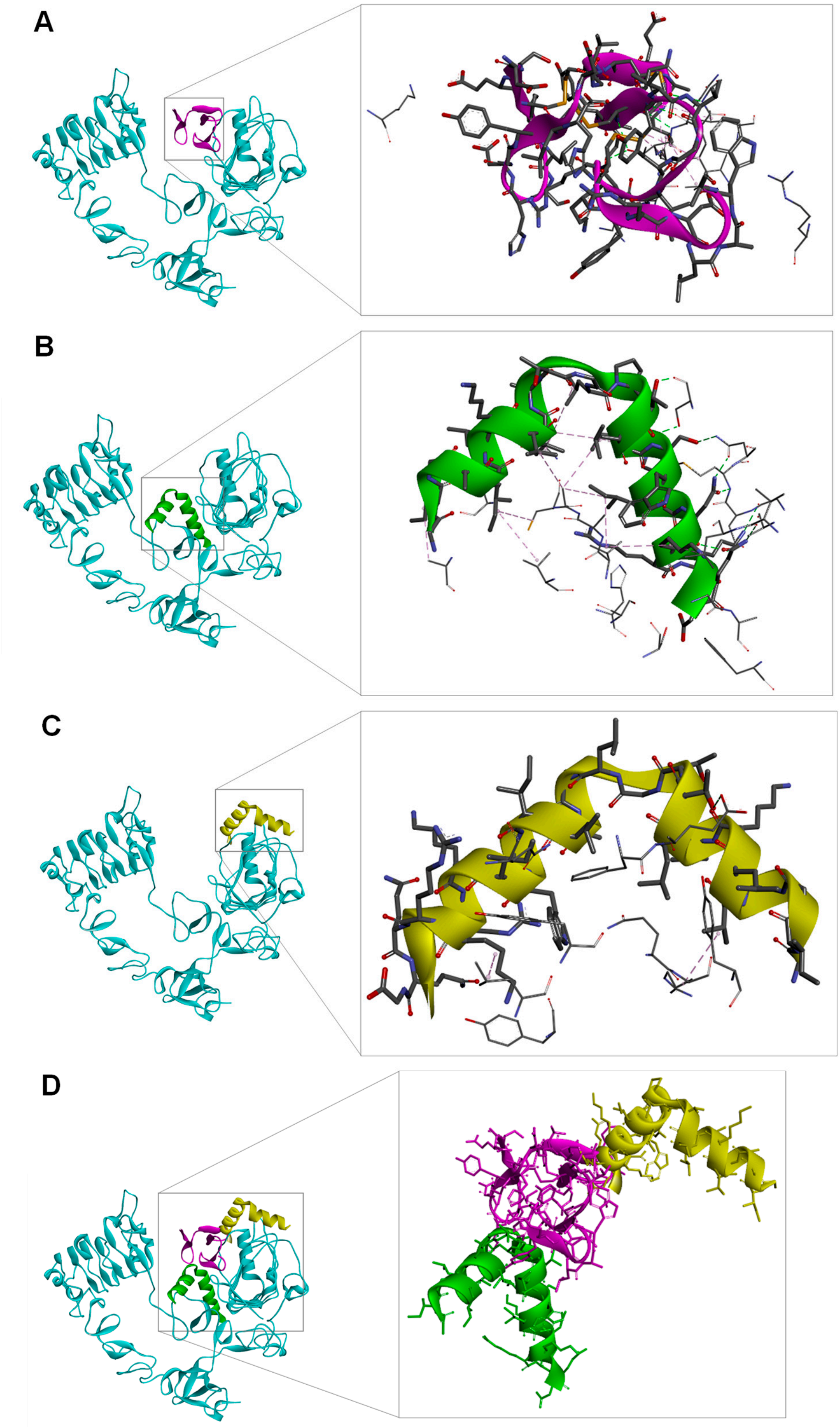

Molecular docking using Z-DOCK (https://zdock.wenglab.org/) was performed comparing between (**A**) EGFR (PDB ID 1NQL) and EGF (**B**) EGFR and Mel-AF and (**C**) EGFR and Mel-AM (**D**) EGFR and EGF, Mel-AF and Mel-AM. The Z scores for top-ranked models were 1239 and 1228 for Mel-AF and Mel-AM, respectively. The left panels show docking sites, and the right panels show the interaction of each ligand to the receptor and the location between each other.

Figure S9. Cytotoxicity of gefitinib on NCI-H1650.

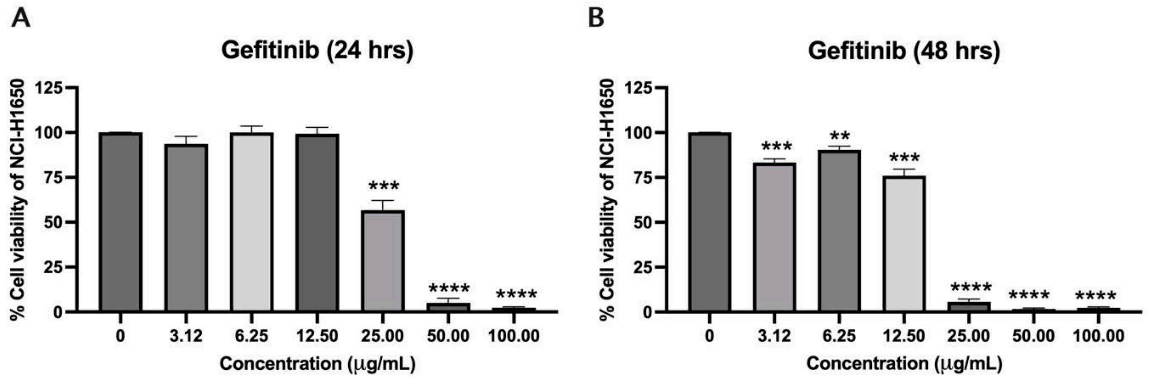

Cytotoxicity of gefitinib was investigated on NCI-H1650 after (**A**) 24 h and (**B**) 48 h of treatment. Data are presented as mean ± SD values of at least three independent replicates. Asterisk (*) indicates significance at $p \leq 0.01$ (**), $p \leq 0.001$ (***) and $p \leq 0.0001$ (****).
